# Antibiotics in Crab Ponds of Lake Guchenghu Basin, China: Occurrence, Temporal Variations, and Ecological Risks

**DOI:** 10.3390/ijerph15030548

**Published:** 2018-03-19

**Authors:** Wenxia Wang, Xiaohong Gu, Lijun Zhou, Huihui Chen, Qingfei Zeng, Zhigang Mao

**Affiliations:** 1 State Key Laboratory of Lake Science and Environment, Nanjing Institute of Geography and Limnology, Chinese Academy of Sciences, Nanjing 210008, China; wangwenxia00@163.com (W.W.); smilingapplewwx@163.com (L.Z.); hhchen@niglas.ac.cn (H.C.); qfzeng@niglas.ac.cn (Q.Z.); zgmao@niglas.ac.cn (Z.M.); 2 University of Chinese Academy of Sciences, Beijing 100049, China

**Keywords:** antibiotics, crab farming, Lake Guchenghu, temporal variation, risk assessment

## Abstract

Antibiotics are widely used in aquaculture, however, this often results in undesirable ecological effects. To evaluate the occurrence, temporal variations, and ecological risk of antibiotics in five crab ponds of Lake Guchenghu Basin, China, 44 antibiotics from nine classes were analyzed by rapid resolution liquid chromatography-tandem mass spectrometry (RRLC-MS/MS). Twelve antibiotics belonging to six classes were detected in the aqueous phase of five crab ponds, among which sulfonamides and macrolides were the predominant classes, and six compounds (sulfamonomethoxine, sulfadiazine, trimethoprim, erythromycin-H_2_O, monensin, and florfenicol) were frequently detected at high concentrations. In general, the antibiotic levels varied between different crab ponds, with the average concentrations ranging from 122 to 1440 ng/L. The antibiotic concentrations in crab ponds exhibited obvious seasonal variations, with the highest concentration and detection frequency detected in summer. Multivariate analysis showed that antibiotic concentrations were significantly correlated with environmental variables, such as total organic carbon, phosphate, ammonia nitrogen, and pH. Sulfadiazine, clarithromycin, erythromycin-H_2_O, and ciprofloxacin posed a high risk to algae, while the mixture of antibiotics could pose a high risk to aquatic organisms in the crab ponds. Overall, the usage of antibiotics in farming ponds should be comprehensively investigated and controlled to preserve a healthy aquaculture ecosystem.

## 1. Introduction

As a new class of environmental contaminants, antibiotics have been the subject of numerous discussions due to their large-scale usage and long-term adverse effects [1,2,3]. Thousands of tons of antibiotics are used for therapeutic purpose or as animal growth promoters every year [4,5,6]. According to market sales data, China is the largest producer and user of antibiotics in the world [7]. In 2013, approximately 162,000 tonnes of antibiotics were used in China, which represents one quarter of the global consumption [1,7]. However, antibiotics administered in aquaculture are rarely absorbed by the target organisms, and approximately 70–80% are excreted via urine and feces [8], leading to the occurrence of antibiotics in many environmental media, such as rivers, lakes, bays, and harbors [1,9,10,11,12].

Previous studies indicated that effluent from farming ponds was one of the main pathways of antibiotics in many water bodies [13,14,15]. With increasing demand for aquatic products, intensive aquaculture methods have been implemented that use antibiotics in order to prevent the emergence of infectious diseases [16,17]. Farming ponds are important aquatic systems in people’s daily lives, however, limited research has been conducted on the residues of antibiotics. Song et al. investigated the antibiotic levels of 24 fishponds in Lake Taihu Basin, and elucidated that antibiotics were at ng/L levels in aqueous phase and detrimental to the growth of algae [18]. Furthermore, the antibiotic levels varied with sampling time, region, and fish species due to the differences in antibiotic usage [7,18]. As a result, comprehensive studies should be conducted focusing on different breeding species, and the potential side effects of antibiotics should be evaluated to ensure a healthy aquaculture ecosystem.

Yang et al. found that pharmaceutical levels were strongly related to the nutrient contents (such as total nitrogen and total phosphorus) of natural water bodies that receive pharmaceuticals from wastewaters with high nutrient content [19]. However, the correlation between antibiotic concentrations and environmental parameters in farming ponds has not been well explored. Pollution sources and hydrographic features may differ between natural water bodies and farming ponds [18], therefore, the correlations between antibiotics and environmental parameters need to be further studied to better understand the potential sources and environmental fate of antibiotics.

Lake Guchenghu is well-known in China for Chinese mitten crab culture, with a total production of 8 × 10^5^ tons in 2016 [20]. Lake Guchenghu is the main water source for aquaculture, and it receives effluent from crab ponds. In this study, the occurrence and distribution of 44 antibiotics from nine classes in the aqueous phase of five crab ponds in Lake Guchenghu Basin were investigated during three breeding seasons. The main objectives of this study were to: (1) better understand the occurrence and temporal variations of antibiotic concentrations in the crab ponds; (2) evaluate the correlation between antibiotic contents and water quality parameters; and (3) assess the ecological risk of antibiotics to aquatic organisms and their detrimental effects on phytoplankton. The resulting data will facilitate the assessment on eco-environmental effects of antibiotics and provide a theoretical basis for pollution control measures in aquaculture. In addition, this study will contribute to further research regarding antibiotic contaminations.

## 2. Materials and Methods

### 2.1. Sampling Ponds and Sample Collection

**Figure 1:**
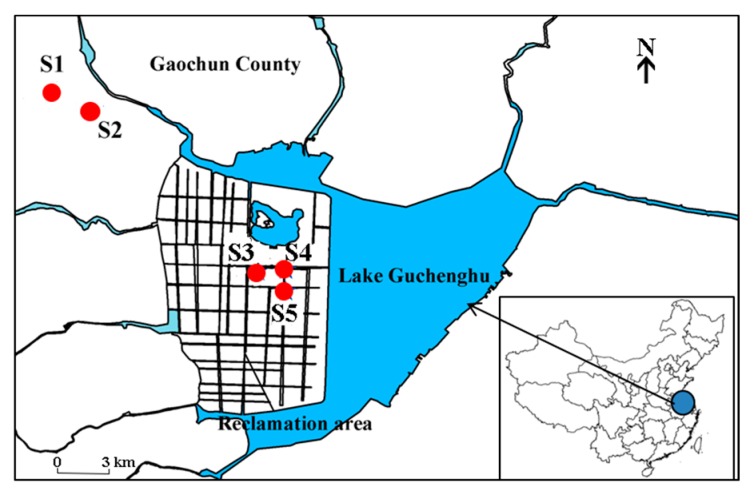
Sample sites in crab ponds around Lake Guchenghu.

Crab ponds surrounding Lake Guchenghu (31°14′~31°18′ N, 118°53′~118°57′ E), which is located in Nanjing City (Jiangsu Province, China), were studied in this investigation. The total surface area of Lake Guchenghu in the 1960s was 65 km^2^, but about half of the area has been reclaimed for commercial crab culture [20]. S3, S4, and S5 are representative crab ponds of the reclaimed area (Figure 1). There is also a large quantity of crab ponds in the upper reaches of Lake Guchenghu, from which S1 and S2 were selected as study sites. 

Surface water was collected from the five crab ponds in May, August, and November, 2016. Duplicate water samples were collected using a water sampler (WB-PM, Beijing Purity Instrument Co., Ltd., Beijing, China), and one liter water samples were stored in brown glass bottles that had been pre-cleaned with methanol. Then the water samples were adjusted to pH 3 using 4 M H_2_SO_4_ and added with methanol (5% v/v) to inhibit microbial activity. Finally, the water samples were kept at 4 °C and target antibiotics were extracted within two days.

### 2.2. Chemicals and Standards

Forty four antibiotics belonging to nine classes were selected based on their usage in human and animals as well as their environmental behaviors. Target antibiotics were sulfonamides (SAs) including sulfacetamide (SCT), sulfachlorpyridazine (SCP), sulfadiazine (SDZ), sulfadoxine (SDO), sulfadimethoxine (SDM), sulfamethazine (SMZ), sulfamethoxazole (SMX), sulfamerazine (SMR), sulfamonomethoxine (SMM), sulfapyridine (SPD), sulfathiazole (STZ), sulfaquinoxaline (SQX), and sulfisoxazole (SX); diaminopyrimidines including trimethoprim (TMP); tetracyclines (TCs) including chlortetracycline (CTC), doxycycline (DC), oxytetracycline (OTC), and tetracycline (TC); fluoroquinolones (FQs) including ciprofloxacin (CFX), danofloxacin (DAN), difloxacin (DIF), enrofloxacin (EFX), fleroxacin (FL), lomefloxacin (LFX), marbofloxacin (MAR), norfloxacin (NFX), ofloxacin (OFX), pefloxacin (PEF), sarafloxacin (SAR), and carbadox (CAR); macrolides (MLs) including azithromycin (ATM), clarithromycin (CTM), erythromycin-H_2_O (ETM-H_2_O), leucomycin (LCM), oleandomycin (ODM), roxithromycin (RTM), and tylosin (TYL); ionophores including salinomycin (SAL), monensin (MON), and narasin (NAR); aminocoumarins including novobiocin (NOV); lincosamides including lincomycin (LIN); and chloramphenicol derivatives, including florfenicol (FF) and chloramphenicol (CAP). More information of the target antibiotics in this study is provided in Table S1.

Oasis hydrophilic-lipophilic balance (HLB) cartridges (6 mL, 500 mg) were supplied by Waters (Milford, MA, USA). Glass fiber filters (GF/F, pore size 0.7 μm) were purchased from Whatman (Maidstone, UK) and pyrolyzed at 450 °C for 4 h prior to use. Methanol and acetonitrile were of HPLC grade and purchased from Merck (Darmstadt, Germany). Other chemicals including disodium ethylenediaminetetraacetate (Na_2_EDTA), citric acid, and sodium citrate were of analytical grade and obtained from Yaohua Chemical Reagent Factory (Tianjin, China).

### 2.3. Sample Preparation and Analysis

Sample extraction and instrumental analyses were performed by following previous methods [13,21]. Solid phase extraction (SPE) was used to extract the water samples. Before extraction, water samples were filtered through glass fiber filters to remove particles. Additionally, 0.2 g Na_2_EDTA and 50 μL surrogate standards (Table S1) were added to each one liter water sample. The HLB cartridges were conditioned with 10 mL methanol and 10 mL Milli-Q water. Then the water samples passed through the cartridges at a speed of 5–10 mL/min and the cartridges were washed with 20 mL Milli-Q water containing 5% methanol and vacuum-dried. Finally, the cartridges were eluted with 3.5 mL methanol for 3 times and the eluent was evaporated to near dryness with a stream of nitrogen at 40 °C, and re-dissolved up to 1 mL with methanol for analysis.

Target antibiotic compounds were analyzed by rapid resolution liquid chromatography–tandem mass spectrometry (RRLC–MS/MS) (Acquity UPLC, Waters, coupled to a Qtrap 5500, AB SCIEX, New York, NY, USA) in the multiple-reaction monitoring (MRM) mode. Two antibiotic compounds (CAP and FF) were analyzed in the negative mode and the other compounds were detected in the positive mode. Target compounds were separated by a BEH-C18 (50 mm × 2.1 mm, 1.7 μm) column equipped with a BEH C18 VanGuard pre-column (5 mm × 2.1 mm, 1.7 μm). The column temperature was 40 °C. The injection volume for each sample was 5 μL. The mobile phases for the positive mode were Milli-Q water with 0.2% formic acid and 2 mM ammonium acetate (A) and acetonitrile (B) at a flow rate of 0.35 mL/min. The mobile phase gradient for the positive mode was ramped from 5% to 10% B in 5 min, 10% to 20% B in 1 min, 20% to 40% B in 3 min, 40% to 50% B in 2 min and ramped to 95% B in 1 min and kept for 12 min. The MS operating conditions in the positive mode were set as follows: curtain gas (CUR), 30; collision gas (CAD), medium; ionSpray voltage (IS), 5500 V; temperature, 550 °C; ion source gas 1, 50; ion source gas 2, 50. The mobile phases for the negative mode were Milli-Q water and acetonitrile at a flow rate of 0.4 mL/min. The mobile phase gradient for the negative mode was ramped from 20% to 40% acetonitrile in 3.2 min, and then ramped to 90% acetonitrile in 0.5 min and kept for 2 min. The MS operating conditions in the negative mode were set as follows: curtain gas (CUR), 30; collision gas (CAD), medium; ionSpray voltage (IS), −4500 V; temperature, 600 °C; ion source gas 1, 60; ion source gas 2, 50. The MS conditions were optimized for collision energy (CE), fragmentor voltage, and MRM transitions for each analyte (Table S1).

### 2.4. Quality Assurance and Quality Control

The internal standard method was used to analyze the concentrations of the target antibiotics. With each set of samples to be analyzed, a solvent blank, a procedure blank, and an independent check standard (100 μg/L standard solutions) were run in sequence to check for carryover, background contamination, and system performance. Appropriate field quality assurance and quality control (QA/QC) procedures were followed. The reported quantitative values of each target compound in the samples were required to have the same retention time as its calibration standard (within 5%) and the same ion ratios (within 20%). An independent check standard was injected approximately every twelve injections, and the concentration computed was required to be within 20% of the expected value. Method detection limits (MDLs) and quantification limits (MQLs) were the minimum detectable amounts of each analyte from the surface water spiked extracts in the MRM mode with signal-to-noise (S/N) of 3 and 10, respectively. The MDLs and MQLs of target antibiotics in the surface water were 0.04–1.22 ng/L and 0.15–4.29 ng/L, respectively. The recoveries of target compounds spiked to the filtered surface water were 45.7–149%.

### 2.5. Risk Characterization

To assess the environmental risk of the antibiotics, two approaches and three trophic levels (algae, daphnia, and fish) were chosen. The first approach is the risk quotient (RQ) of individual compound, which is calculated using the following formula:

RQ = MEC/PNEC(1)

where MEC is the maximum measured environmental concentration, and PNEC is the predicted no effect concentration in water. The PNEC in water is calculated as follows:

PNEC = (LC_50_ or EC_50_)/AF (2)

where LC_50_ or EC_50_ is the lowest median effective concentration value [14], and AF is an appropriate standard assessment factor (1000).

The second approach is to use the mixture risk quotient (MRQ), which is expressed by two parameters, MRQ_MEC/PNEC_ and MRQ_STU_ [22]. STU is the sum of toxic units. The formulas for calculation are as follows:

(3) $${\mathrm{MRQ}}_{\mathrm{STU}}=\max({\mathrm{STU}}_{algae},{\mathrm{STU}}_{daphnids},{\mathrm{STU}}_{fish})\;\times \;\mathrm{AF}=\;\mathrm{Max}(\sum_{i=1}^{n}\frac{{\mathrm{MEC}}_{i}}{{\mathrm{EC}}_{50i},\mathrm{algae}},\sum_{i=1}^{n}\frac{{\mathrm{MEC}}_{i}}{{\mathrm{EC}}_{50i},\mathrm{daphnids}},\sum_{i=1}^{n}\frac{{\mathrm{MEC}}_{i}}{{\mathrm{EC}}_{50i},\mathrm{fish}})\;\times \;\mathrm{AF}$$

(4) $${\mathrm{MRQ}}_{\mathrm{MEC}/\mathrm{PNEC}}=\sum_{i=1}^{n}\frac{{\mathrm{MEC}}_{i}}{{\mathrm{PNEC}}_{i}}\;=\;\sum_{i=1}^{n}\frac{{\mathrm{MEC}}_{i}}{\min{\left({\mathrm{EC}}_{50},\mathrm{algae},\;{\mathrm{EC}}_{50},\mathrm{daphnids},\;{\mathrm{EC}}_{50},\mathrm{fish}\right)}_{i}}\;\times \;{\mathrm{AF}}_{i}$$

where n is the number of target antibiotics. An RQ or MRQ value of higher than 1 indicates a high risk to aquatic organisms, whereas values of 0.1~1 and 0.01~0.1 indicate a medium and low risk, respectively, and the values below 0.01 indicate minimal or no risk to organisms [23].

### 2.6. Environmental Parameters Determination and Statistical Analysis

Environmental parameters, including water transparency (WT), pH, electronic conductivity (EC), dissolved oxygen (DO), total nitrogen (TN), total dissolved nitrogen (TDN), total phosphorus (TP), total dissolved phosphorus (TDP), phosphate (PO_4_^3−^-P), ammonia nitrogen (NH_4_^+^-N), nitrate nitrogen (NO_3_^−^-N), total organic carbon (TOC), and chlorophyll a (Chl-a) were measured in this study. WT, pH, EC, and DO were measured in situ by a YSI system (YSI, Yellow Springs, OH, USA). Chl-a was measured spectrophotometrically from matter retained on a GF/C filter over 24 h and extracted in a 90% (v/v) acetone/water solution. Other environmental parameters including TN, TDN, TP, TDP, PO_4_^3−^-P, NH_4_^+^-N, NO_3_^−^-N, and TOC were determined according to standard laboratory methods [24]. 

Multivariate analyses, including detrended correspondence analysis (DCA) and redundancy analysis (RDA), were used to evaluate the relationship between antibiotic pollution characteristics and various environmental parameters. If the length of the first ordination gradient calculated by DCA is <3, RDA should be used for the data set. Correlations between the antibiotic levels and Chl-a concentrations in the crab ponds of Lake Guchenghu Basin were tested. Pearson correlations were used if the data were normally distributed, while Spearman correlations were used if the data were not normally distributed. Statistical analysis was conducted using IBM SPSS Statistics 22.0 (IBM SPSS Software, Armonk, NY, USA) and Canoco for Windows 4.5 (Microcomputer Power, Issacard, NY, USA). Figures were plotted with Origin software (Version 8.0, Origin Lab., Northampton, MA, USA).

## 3. Results

### 3.1. Antibiotics in Crab Ponds of Lake Guchenghu Basin

**Figure 2:**
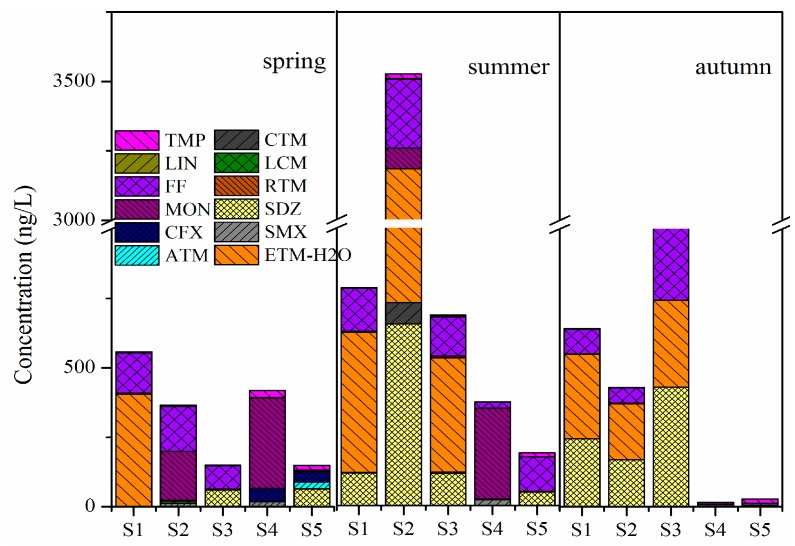
Composition of antibiotics in aqueous phase of crab ponds during three seasons.

The concentrations of antibiotics in the surface water of crab ponds around Lake Guchenghu are summarized in Figure 2. Twelve antibiotics belonging to six classes were detected, with total concentrations ranging from 14.5 to 3520 ng/L (Table S2). Among these compounds, SAs and MLs were the predominant classes and six antibiotics (SMM, SDZ, TMP, ETM-H_2_O, MON and FF) were frequently detected at high concentrations. Significant differences were observed between the antibiotic levels of the ponds, with average concentrations descending in the following order: S2 (1440 ng/L) > S1 (661 ng/L) > S3 (614 ng/L) > S4 (269 ng/L) > S5 (122 ng/L). The antibiotic concentrations of S2 were significantly higher than those of other ponds, among which ETM-H_2_O and SDZ were the predominant compounds. Furthermore, the total antibiotic levels showed obvious seasonal variations, with the highest concentrations detected in summer, followed by autumn and spring.

MLs were detected with a frequency of 100%, among which ETM-H_2_O was the predominant compound with the maximum concentration of 2450 ng/L. In spring, ETM-H_2_O was only detected at S1 with a high concentration, while in summer and autumn, ETM-H_2_O was abundant at S1, S2, and S3. ATM was detected at S5 in spring (24.2 ng/L) and autumn (5.60 ng/L). CTM was detected at S1 (3.20 ng/L), S2 (76.2 ng/L), and S3 (6.30 ng/L) in summer. LCM was only detected at S2 (8.00 ng/L) in spring, and RTM was only detected in autumn with low concentrations.

For SAs, SMX was detected at S2 in spring (12.5 ng/L), and S4 in spring (17.5 ng/L), summer (21.7 ng/L), and autumn (6.40 ng/L). In summer and autumn, SDZ was detected at all sites excluding S4, and the highest concentration was observed at S2 (654 ng/L), while in spring it was detected at S3 (59.4 ng/L) and S5 (63.2 ng/L). The sulfonamide potentiator TMP was detected with concentrations ranging from ND (not detected) to 24.5 ng/L.

In spring and autumn, FF was detected at S1, S2, and S3, while in summer it was detected at all the sampling sites. MON was detected at S4 in spring (326 ng/L) and summer (328 ng/L), and S2 in spring (177 ng/L). CFX was detected at S4 (46.5 ng/L) and S5 (37.6 ng/L) in spring. LIN was frequently detected at low concentrations (ND—4.40 ng/L).

### 3.2. Relationship between Antibiotics and Environmental Parameters

The correlation between antibiotic distribution and environmental parameters (i.e., water quality parameters) was evaluated by multivariate analysis. DCA indicated that the length of the gradient in the first axis was 2.080; hence, the RDA model was used [25].

**Figure 3:**
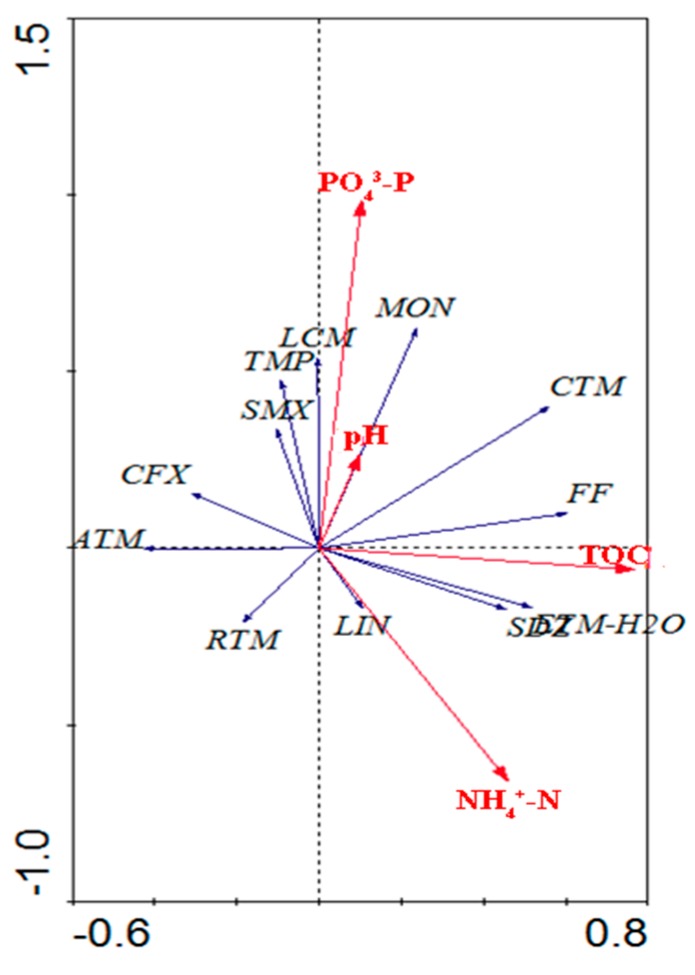
Canonical correspondence analysis of the antibiotic concentrations and environmental parameters in surface water of crab ponds.

The correlation between antibiotic concentrations and environmental variables with the first two axes of RDA is shown in Figure 3. The first and second axes of the percentage variance of the species-environment relation revealed by RDA were 50.8% and 27.0% for the water samples (Table S3). RDA indicated that 39% of the variation was explained by significant environmental variables including TOC (0.121), PO_4_^3−^-P (0.104), NH_4_^+^-N (0.092), and pH (0.08). The results showed a significant contribution (*p* < 0.05), which indicated that the distribution and concentrations of antibiotics in the aqueous phase were strongly correlated with these environmental variables.

### 3.3. Risk Assessment

**Figure 4:**
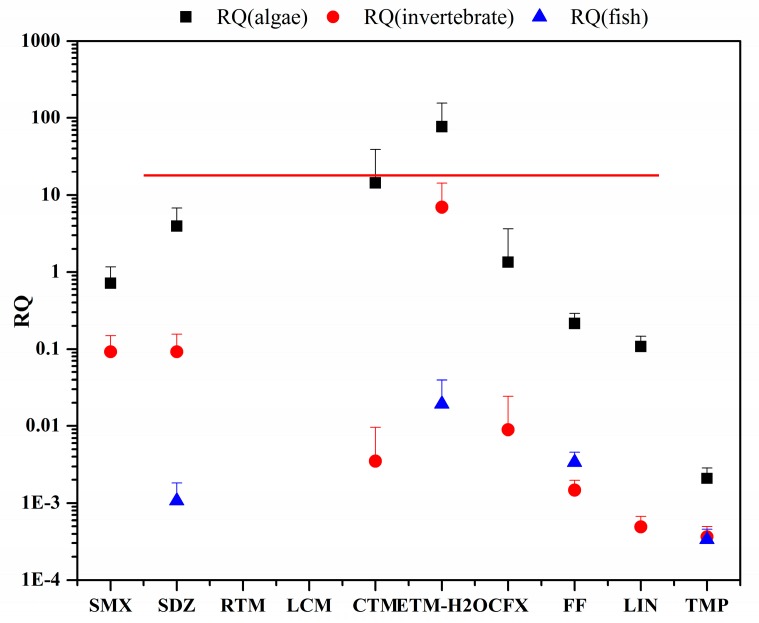
Individual RQ values of antibiotics in ponds of Lake Guchenghu Basin.

The risk quotient (RQ) of antibiotics to organisms is presented in Figure 4. The RQ values of SDZ, CTM, ETM-H_2_O, and CFX for algae were over 1, indicating that these antibiotics may pose a high risk to algae in these ponds. 

The RQ values of SMX, FF, and LIN to algae ranged from 0.11 to 0.72, suggesting that they posed a medium risk. Additionally, the RQ value of ETM-H_2_O for invertebrates was over 1, indicating that it posed a high risk. The RQ values of RTM and LCM were below 0.01, which means that they may not pose a risk to organisms in this study. 

Similar values ranging from 26.9 to 218 were found for MRQ_MEC/PNEC_ and MRQ_STU_, indicating that the target antibiotics could pose a high risk to aquatic organisms in the ponds. The high MRQ values were mainly caused by the high individual RQ values of ETM-H_2_O and CTM. The correlation between the total antibiotic levels and Chl-a concentration in the surface water of the crab ponds in Lake Guchenghu Basin was tested. The results showed that the total antibiotic levels were not significantly related to the Chl-a concentrations (Spearman correlation coefficient of 0.343, *P* = 0.211).

## 4. Discussion

### 4.1. Pollution Levels of Antibiotics in Crab Ponds of Lake Guchenghu Basin

Antibiotics were abundant in the crab ponds of Lake Guchenghu Basin as they are widely used to prevent the prevalence of bacterial diseases among aquaculture species. Previous studies indicated that the occurrence of antibiotics varied in different breeding species as different antibiotics were administered [18]. There were different pollution levels between the five ponds, which may be due to differences in cultivation patterns [18,26]. The antibiotic concentrations in crab ponds were slightly lower than those of fish and shrimp ponds in previous studies [27,28], which may be because antibiotics are less used in crab ponds than in fish and shrimp ponds. The antibiotic concentrations in mariculture areas were in consistent with those detected at S4 and S5, but lower than those at S1, S2 and S3. This was because unlike crab ponds, mariculture areas are often semi-enclosed, and antibiotics are more easily diluted and washed away [29,30]. 

Water bodies with larger areas often have more contamination sources, including agriculture, animal husbandry, and domestic wastewater. However, the antibiotic levels measured in this study were higher than those in most lakes and rivers, while they are consistent with those reported in seriously polluted areas, such as the Pearl Estuary [11], Baiyangdian Lake [22], and Hai River [29]. This may be because a large amount of antibiotics are used in ponds during aquaculture.

In this study, six antibiotics (ETM-H_2_O, SDZ, FF, MON, SMM, and TMP) were frequently detected at high concentrations. The concentration of ETM-H_2_O in the crab ponds was higher than that in Lake Taihu [13], the Pearl River [11], Yellow River [30], and streams in Jianghan Plain [14]. ETM-H_2_O is the degradation of erythromycin, which is effective in the prevention and treatment of various tissue infections, and has been extensively used since its introduction in 1952 [31]. Additionally, ETM-H_2_O has high solubility and chemical stability in aquatic environments [32]. The high levels of ETM-H_2_O in this study may be the result of extensive usage as well as its physicochemical properties. SDZ was frequently detected in Lake Chaohu [32], Lake Baiyangdian [22], and the Pearl River [11] with concentrations ranging from ND—45.6, 0.86–505, and ND—336 ng/L, respectively, which was in consistent with that measured in this study. FF was detected at much lower concentrations in other areas [33,34], excluding Lake Taihu, which had a maximum concentration of 963 ng/L [13]. SMM, SDZ, and FF are typical medicines in aquaculture, with total usages of 2210, 1260 and 10,000 tons, respectively, in China in 2013 [7]. In addition, obvious seasonal variations were observed, which largely reflected the high utilization rates during disease outbreaks. The ionophore MON was not frequently detected in China, but it was found in six Australian rivers (ND—150 ng/L) [35]. Other compounds, such as ROX, were not detected in this study as they are human-derived and are often detected in domestic wastewater [36]. The relatively low concentrations of FQs in this study may because they degrade easily and are not extensively used [37]. Overall, the composition and spatiotemporal variations of antibiotics indicated that antibiotics in farming ponds were affected by their sources and physicochemical properties, such as water solubility and degradability.

### 4.2. Temporal Variations of Antibiotics and Correlative Environmental Factors in Crab Ponds

The highest antibiotic concentrations were detected in summer, which was related to crab breeding activity. In summer, diseases may outbreak more easily due to the high temperature. Antibiotics were administrated to prevent and cure diseases, a significant fraction of which ended up in the aquatic environment by the parent forms or their active metabolites [38]. In autumn, antibiotic concentrations decreased due to attenuation processes, such as degradation, adsorption, and infiltration. The results were contrary to studies where antibiotics were the lowest in summer when the degradation of antibiotics occurred more readily due to the high temperature [39,40]. However, continuous introduction of antibiotics may play a more important role in the antibiotic levels of crab ponds. Significant temporal variations were observed in compounds such as ETM-H_2_O, SDZ, and CTM, which are widely used in aquaculture activities [7].

Multivariate analysis showed that the antibiotic concentrations in crab ponds were related to environmental parameters such as TOC, PO_4_^3−^-P, NH_4_^+^-N, and pH. The correlation between antibiotic concentrations and nutrient levels may be due to similarities in their sources. In farming ponds, the increase in nutrient levels including PO_4_^3−^-P and NH_4_^+^-N may result from extensive feed [20], so medicated feed may also influence the antibiotic levels of crab ponds.

Antibiotics may exhibit different environmental behaviors (such as discharge, infiltration, adsorption, and degradation) in aquatic environments [23]. Environmental factors, such as pH, may influence the concentrations of antibiotics by altering their environmental fate. Previous studies indicated that sediment was an important sink for antibiotics, and adsorption was largely affected by the TOC contents of the sediment and water [13]. As a portion of the TOC, the content of DOC was affected by the microbial and photo-degradation. The bacteria involved in this process may also affect antibiotic degradation [41]. Overall, the antibiotics’ physicochemical properties, pollution sources, and environmental parameters such as pH and TOC could be important factors that influence the levels and distributions of antibiotics.

### 4.3. Environmental Application

The risk assessment suggested that SDZ, CTM, ETM-H_2_O, and CFX may pose a high risk to the algae in the ponds. If the effects of mixture antibiotics were considered, the ecological risk would be higher. Changes in algal communities may influence the aquatic ecosystem and pose a potential risk to aquatic organisms [42]. This study demonstrated that the antibiotic concentrations in the ponds were not significantly related to algal density (measured as Chl-a), which was because the growth of algae was also influenced by other factors such as water parameters and hydrological conditions [43]. Antibiotics may accumulate in aquatic products through long-term exposure or the food chain, which would not only affect our metabolism and normal intestinal flora, but also potentially exert negative effects on the spread and dissemination of antibiotic resistance genes [44]. Zhang et al. reported a positive correlation between antibiotic-resistant *Escherichia coli* numbers and corresponding antibiotic levels [45]. More seriously, previous studies have denoted that aquaculture is one of the main sources of antibiotics in rivers and lakes as wastewater from farming ponds may be released into the receiving aquatic environment [21].

Crabs are one of the most popular aquatic products for human consumption in China as well as other Asian countries, and the crab ponds of Lake Guchenghu Basin are an important breeding base. This study showed that antibiotics, especially SDZ, CTM, ETM-H_2_O, and CFX, are overused in crab ponds. Antibiotics may enter ponds through several pathways, including direct splashing, medicated feed as well as medicated bath [18] and the usage of drugs is a sensitive and private problem, which makes it difficult to determine the input of antibiotics in farming ponds. As medicated feed may influence antibiotic residues, the input of antibiotics to farming ponds should be comprehensively assessed and controlled. Farmers commonly lack the knowledge required to use antibiotics rationally, so the restrictions on antibiotic usage should be enforced according to pollution sources, ecological risk, and the antibiotics’ physicochemical properties. In addition, comprehensive evaluation regarding antibiotic contamination and its potential side effects in the aquatic environment is necessary.

## 5. Conclusions

In this study, the occurrence and distribution of antibiotic residues in the crab ponds of Lake Guchenghu Basin were investigated during three breeding seasons. Antibiotics were abundant in the crab ponds, and the concentrations were slightly lower than those documented in fish and shrimp ponds. Six antibiotics (SMM, SDZ, TMP, ETM-H_2_O, MON, and FF) were detected frequently at high concentrations. The highest antibiotic concentrations were detected in summer, which resulted from the introduction of antibiotics in aquaculture. Multivariate analysis showed that the antibiotic concentrations of crab ponds were related to environmental parameters such as TOC, PO_4_^3−^-P, NH_4_^+^-N and pH. The risk assessment based on the RQ values showed that SDZ, CTM, ETM-H_2_O, and CFX posed a high risk to algae, and the mixture of antibiotics could be detrimental to the aquatic organisms in crab ponds. Overall, the antibiotic concentrations in crab ponds were affected by pollution sources, their physicochemical properties as well as environmental factors, and the antibiotic residues should be investigated and controlled to preserve a healthy aquaculture ecosystem.

## Supplementary Materials

The following are available online at http://www.mdpi.com/1660-4601/15/3/548/s1, Table S1: Details of antibiotics and their MRM parameters in RRLC-MS/MS, Table S2: Concentrations of antibiotics in ponds around Lake Guchenghu (ng/L), Table S3: Canonical correspondence analysis of the antibiotic concentrations and environmental parameters in crab ponds.

## References

[1] Archundia D., Duwig C., Lehembre F., Chiron S., Morel M.C., Prado B., Bourdat-Deschamps M., Vince E., Flores Aviles G., Martins J.M.F. (2017). Antibiotic pollution in the Katari subcatchment of the Titicaca Lake: Major transformation products and occurrence of resistance genes. Sci. Total Environ..

[2] Berglund B., Khan G.A., Lindberg R., Fick J., Lindgren P.E. (2014). Abundance and Dynamics of Antibiotic Resistance Genes and Integrons in Lake Sediment Microcosms. PLoS ONE.

[3] Hladicz A., Kittinger C., Zarfel G. (2017). Tigecycline Resistant *Klebsiellapneumoniae* Isolated from Austrian River Water. Int. J. Environ. Res. Public Health.

[4] Kümmerer K. (2009). Antibiotics in the Aquatic Environment—A Review—Part I. Chemosphere.

[5] Kümmerer K. (2009). Antibiotics in the Aquatic Environment—A Review—Part II. Chemosphere.

[6] Sarmah A.K., Meyer M.T., Boxall A.B.A. (2006). Global Perspective on the Use, Sales, Exposure Pathways, Occurrence, Fate and Effects of Veterinary Antibiotics (VAs) in the Environment. Chemosphere.

[7] Zhang Q.Q., Ying G.G., Pan C.G., Liu Y.S., Zhao J.L. (2015). Comprehensive Evaluation of Antibiotics Emission and Fate in the River Basins of China: Source Analysis, Multimedia Modeling, and Linkage to Bacterial Resistance. Environ. Sci. Technol..

[8] Bound J.P., Voulvoulis N. (2004). Pharmaceuticals in the Aquatic Environment––A Comparison of Risk Assessment Strategies. Chemosphere.

[9] Cui C.Z., Jin L., Jiang L., Han Q., Lin K.F., Lu S.G., Zhang D., Cao G.M. (2016). Removal of Trace Level Amounts of Twelve Sulfonamides from Drinking Water by UV-activated Peroxymonosulfate. Sci. Total Environ..

[10] Murata A., Takada H., Mutoh K., Hosoda H., Harada A., Nakada N. (2011). Nationwide Monitoring of Selected Antibiotics: Distribution and Sources of Sulfonamides, Trimethoprim, and Macrolides in Japanese Rivers. Sci. Total Environ..

[11] Yang J.F., Ying G.G., Zhao J.L., Tao R., Su H.C., Chen F. (2010). Simultaneous Determination of Four Classes of Antibiotics in Sediments of the Pearl Rivers Using RRLC–MS/MS. Sci. Total Environ..

[12] Gibs J., Heckathorn H.A., Meyer M.T., Klapinski F.R., Alebus M., Lippincott R.L. (2013). Occurrence and Partitioning of Antibiotic Compounds Found in the Water Column and Bottom Sediments from a Stream Receiving Two Wastewater Treatment Plant Effluents in Northern New Jersey, 2008. Sci. Total Environ..

[13] Zhou L.J., Wu Q.L., Zhang B.B., Zhao Y.G., Zhao B.Y. (2016). Occurrence, Spatiotemporal Distribution, Mass Balance and Ecological Risks of Antibiotics in Subtropical Shallow Lake Taihu, China. Environ. Sci. Process. Impacts.

[14] Yao L., Wang Y., Tong L., Deng Y., Li Y., Gan Y., Guo W., Dong C., Duan Y., Zhao K. (2017). Occurrence and Risk Assessment of Antibiotics in Surface Water and Ground Water from Different Depths of Aquifers: A Case Study at Jianghan Plain, Central China. Ecotoxicol. Environ. Saf..

[15] Chen H., Liu S., Xu X.R., Zhou G.J., Liu S.S., Yue W.Z. (2015). Antibiotics in the Coastal Environment of the Hailing Bay Region, South China Sea: Spatial Distribution, Source Analysis and Ecological Risks. Mar. Pollut. Bull..

[16] Kim S.C., Carlson K. (2007). Temporal and Spatial Trends in the Occurrence of Human and Veterinary Antibiotics in Aqueous and River Sediment matrices. Environ. Sci. Technol..

[17] Cai C.F., Ye Y.T., Chen L.Q., Qin J.G., Wang Y.L. (2010). Oxygen consumption and ammonia excretion of black carp (*Mylopharyngdon piceus* Richardson) and allogynogenetic crucian carp (*Carassius auratus gibelio* ♀ × *Cyprinus carpio* ♂) fed different carbohydrate diets. Fish Physiol. Biochem..

[18] Song C., Zhang C., Fan L.M., Qiu L.P., Wu W., Meng S.L., Hu G.D., Kamira B., Chen J.Z. (2016). Occurrence of Antibiotics and Their Impacts to Primary Productivity in Fishponds around Tai Lake, China. Chemosphere.

[19] Yang X., Chen F., Meng F.G., Xie Y.Y., Chen H., Young K., Luo W.X., Ye T.J., Fu W.J. (2013). Occurrence and Fate of PPCPs and Correlations with Water Quality Parameters in Urban Riverine Waters of the Pearl River Delta, South China. Environ. Sci. Pollut. Res..

[20] Zeng Q.F., Gu X.H., Chen X., Mao Z.G. (2013). The Impact of Chinese Mitten Crab Culture on Water Quality, Sediment and the Pelagic and Macrobenthic Community in the Reclamation Area of Guchenghu Lake. Fish Sci..

[21] Zhou L.J., Ying G.G., Liu S., Zhao J.L., Chen F., Zhang R.Q., Peng F.Q., Zhang Q.Q. (2012). Simultaneous Determination of Human and Veterinary Antibiotics in Various Environmental Matrices by Rapid Resolution Liquid Chromatography–Electrospray Ionization Tandem Mass Spectrometry. J. Chromatogr. A..

[22] Li W.H., Shi Y.L., Gao L.H., Liu J.M., Cai Y.Q. (2012). Occurrence of Antibiotics in Water, Sediments, Aquatic Plants, and Animals from Baiyangdian Lake in North China. Chemosphere.

[23] Yan C., Yang Y., Zhou J., Liu M., Nie M., Shi H., Gu L. (2013). Antibiotics in the Surface Water of the Yangtze Estuary: Occurrence, Distribution and Risk assessment. Environ. Pollut..

[24] American Public Health Association (APHA) (2005). Standard Methods for the Examination of Water and Wastewater.

[25] LepŠ J., Šmilauer P. (2003). Multivariate Analysis of Ecological Data Using CANOCO.

[26] Xuan L.T., Munekage Y. (2004). Residues of Selected Antibiotics in Water and Mud from Shrimp Ponds in Mangrove Areas in VietNam. Mar. Pollut. Bull..

[27] Romero-González R., López-Martínez J.C., Gómez-Milán E., Garrido-Frenich A., Martínez-Vidal J.L. (2007). Simultaneous Determination of Selected Veterinary Antibiotics in Gilthead Seabream (sparusaurata) by Liquid Chromatography–Mass Spectrometry. J. Chromatogr. B..

[28] Na G.S., Fang X.D., Cai Y.Q., Ge L.K., Zong H.M., Yuan X.T., Yao Z.W., Zhang Z.F. (2013). Occurrence, Distribution, and Bioaccumulation of Antibiotics in Coastal Environment of Dalian, China. Mar. Pollut. Bull..

[29] Luo Y., Xu L., Rysz M., Wang Y., Zhang H., Alvarez P.J.J. (2011). Occurrence and Transport of Tetracycline, Sulfonamide, Quinolone, and Macrolide Antibiotics in the Haihe River Basin, China. Environ. Sci. Technol..

[30] Zhang R., Tang J., Li J., Cheng Z., Chaemfa C., Liu D., Zheng Q., Song M., Luo C., Zhang G. (2013). Occurrence and Risks of Antibiotics in the Coastal Aquatic Environment of the Yellow Sea, North China. Sci. Total Environ..

[31] Wang J. (2009). Analysis of macrolide antibiotics, using liquid chromatography-mass spectrometry, in food, biological and environmental matrices. Mass Spectrom. Rev..

[32] Tang J., Shi T.Z., Wu X.W., Cao H.Q., Li X.D., Hua R.M., Tang F., Yue Y.D. (2015). The Occurrence and Distribution of Antibiotics in Lake Chaohu, China: Seasonal Variation, Potential Source and Risk Assessment. Chemosphere.

[33] Hoa P.T., Managaki S., Nakada N., Takada H., Shimizu A., Anh D.H., Viet P.H., Suzuki S. (2011). Antibiotic Contamination and Occurrence of Antibiotic-resistant Bacteria in Aquatic Environments of Northern Vietnam. Sci. Total Environ..

[34] Bai Y.W., Meng W., Xu J., Zhang Y., Guo C.S. (2014). Occurrence, Distribution and Bioaccumulation of Antibiotics in the Liao River Basin in China. Environ. Sci. Process. Impacts.

[35] Hirsch R., Ternes T., Haberer K., Kratz K.L. (1999). Occurrence of Antibiotics in the Aquatic Environment. Sci. Total Environ..

[36] Xu J., Zhang Y., Zhou C.B., Guo C.S., Wang D.M., Du P., Luo Y., Wan J., Meng W. (2014). Distribution, Sources and Composition of Antibiotics in Sediment, Overlying Water and Pore Water from Taihu Lake, China. Sci. Total Environ..

[37] Pico Y., Andreu V. (2007). Fluoroquinolones in Soil-Risks and Challenges. Anal. Bioanal. Chem..

[38] Matyar F., Gulnaz O., Guzeldag G., Mercimek H.A., Akturk S., Arkut A., Sumengen M. (2014). Antibiotic and Heavy Metal Resistance in Gram-negative Bacteria Isolated from the Seyhan Dam Lake and Seyhan River in Turkey. Ann. Microbiol..

[39] Kolpin D.W., Furlong E.T., Meyer M.T., Thurman E.M., Zaugg S.D., Barber L.B., Buxton H.T. (2002). Pharmaceuticals, Hormones, and other Organic Waste Contaminants in U.S. streams, 1999–2000: A National Reconnaissance. Environ. Sci. Technol..

[40] Karthikeyan K.G., Meyer M.T. (2006). Occurrence of Antibiotics in Wastewater Treatment Facilities in Wisconsin, USA. Sci. Total Environ..

[41] He B.Y., Dai M.H., Zhai W.D., Wang L.F., Wang K.J., Chen J.H., Lin J.R., Han A.Q., Xu Y.P. (2010). Distribution, Degradation and Dynamics of Dissolved Organic Carbon and Its Major Compound Classes in the Pearl River Estuary, China. Mar. Chem..

[42] Wu T.F., Qin B.Q., Zhu G.W., Luo L.C., Ding Y.Q., Bian G.Y. (2013). Dynamics of Cyanobacterial Bloom Formation during Short-term Hydrodynamic Fluctuation in a Large Shallow, Eutrophic, and Wind-exposed Lake Taihu, China. Environ. Sci. Pollut. Res..

[43] Paerl H.W., Hall N.S., Calandrino E.S. (2011). Controlling Harmful Cyanobacterial Blooms in a World Experiencing Anthropogenic and Climatic-induced Change. Sci. Total Environ..

[44] Cabello F.C., Godfrey H.P., Tomova A., Ivanova L., Dolz H., Millanao A., Buschmann A.H. (2013). Antimicrobial Use in Aquaculture Re-examined: Its Relevance to Antimicrobial Resistance and to Animal and Human Health. Environ. Microbiol..

[45] Zhang Q., Jia A., Wan Y., Liu H., Wang K., Peng H., Dong Z., Hu J. (2014). Occurrences of Three Classes of Antibiotics in a Natural River Basin: Association with Antibiotic-resistant *Escherichia coli*. Environ. Sci. Technol..

