# Supplementary material for: Antibiotics in Crab Ponds of Lake Guchenghu Basin, China: Occurrence, Temporal Variations, and Ecological Risks
Source: Int J Environ Res Public Health. 2018 Mar 19;15(3):548. doi: 10.3390/ijerph15030548 (PMC5877093; doi:10.3390/ijerph15030548)
Supplement: Supplementary file 1 [file ijerph-15-00548-s001.pdf]

International Journal of
Environmental Research and Public Health

MDPI

Supplementary information

Antibiotics in the Crab Ponds of Lake Guchenghu Basin, China: Occurrence, Temporal Variations and Ecological Risks

Wenxia Wang[1, 2],Xiaohong Gu[1,*],Lijun Zhou[1],Huihui Chen[1],Qingfei Zeng[1],Zhigang Mao[1]

[1] State Key Laboratory of Lake Science and Environment, Nanjing Institute of Geography and Limnology, Chinese Academy of Sciences, Nanjing 210008, China; wangwenxia00@163.com (W.W.); smilingapplewwx@163.com (L.Z.); hhchen@niglas.ac.cn (H.C.); qfzeng@niglas.ac.cn (Q.Z.); zgmao@niglas.ac.cn (Z.M.)

[2] University of Chinese Academy of Sciences, Beijing 100049, China

* Correspondence: xhgu@niglas.ac.cn; Tel.: +86-025-8688-2006

Table S1 Details of antibiotics and their MRM parameters in RRLC-MS/MS

Class	Compound	Abbr [a]	CAS	Supplier	R.T.[b] (min)	MRM-transitions[c]	Fragmentor (V)	CE[d] (eV)
**ESI+**								
**Sulfonamides**		**SAs**						
	Sulfacetamide	SCT	144-80-9	Dr.Ehrenstorfer	0.79	215→91.8	40	28
						215→155.9	40	14
	Sulfachloryridazine	SCP	80-32-0	Dr.Ehrenstorfer	2.79	284.8→155.8	40	19
						284.8→107.9	40	32
	Sulfadiazine	SDZ	68-35-9	Dr.Ehrenstorfer	0.91	250.9→155.7	40	20
						250.9→92	40	32
	Sulfadoxine	SDO	2447-57-6	Dr.Ehrenstorfer	3.51	310.9→155.9	60	26
						310.9→107.9	60	33
	Sulfadimethoxine	SDM	122-11-2	Dr.Ehrenstorfer	5.86	310.8→155.9	100	27
						310.8→107.9	100	33
	Sulfamethazine	SMZ	57-68-1	Dr.Ehrenstorfer	1.83	279→185.9	75	23
						279→155.9	75	25
	Sulfathiazole	STZ	72-14-0	Dr.Ehrenstorfer	1.06	256→155.8	90	18
						256→91.8	90	30
	Sulfamethoxazole	SMX	723-46-6	Dr.Ehrenstorfer	3.42	253.9→155.9	90	22
						253.9→107.9	90	30
	Sulfamethoxazole-D_4 (IS) [e]	SMX-D_4	1020719-86-1	Dr.Ehrenstorfer	3.36	258.1→159.9	90	20
						258.1→111.9	90	32
	Sulfamerazine	SMR	127-79-7	Dr.Ehrenstorfer	1.31	264.8→92.1	70	34
						264.8→108.2	70	33
	Sulfamerazine-D_4 (IS)	SMR-D_4		TRC	1.28	269→159.9	70	21
						269→109.8	70	27
	Sulfamonomethoxine	SMM	1220-83-3	Dr.Ehrenstorfer	2.7	281.2→155.8	110	24
						281.2→107.8	110	31
	Sulfapyridine	SPD	144-83-2	Dr.Ehrenstorfer	1.45	249.9→155.9	60	21
						249.9→92.1	60	33
	Sulfaquinoxaline	SQX	59-40-5	Dr.Ehrenstorfer	6.11	300.9→91.8	90	31
						301.3→107.9	90	32
	Sulfisoxazole	SX	127-69-5	Dr.Ehrenstorfer	4.15	268→156.0	90	17
						268→112.9	90	21
**diaminopyrimidines**	Trimethoprim	TMP	738-70-5	Dr.Ehrenstorfer	1.71	291.1→122.9	150	31
						291.2→230.1	150	31
	Trimethoprim-D_3 (IS)	TMP- D_3		TRC	1.66	294.4→123.1	150	30
						294.4→230.0	150	47
**Tetracyclines**		TCs						
	Chlortetracycline	CTC	57-62-5	Dr.Ehrenstorfer	5.08	479→444	90	27
						479→462.1	90	23
	Doxycycline	DC	564-25-0	Dr.Ehrenstorfer	6.5	445→428.1	155	22
						445→410	155	32
	Thiabendazole-D_4 (IS)	TBD-D_4	71535-97-2	TRC	1.09	205.9→134.9	180	45
						205.9→178.9	180	35
	Oxytetracycline	OTC	79-57-2	Dr.Ehrenstorfer	1.94	461.1→426.1	80	25
						461.1→443.1	80	17
	Tetracycline	TC	60-54-8	Dr.Ehrenstorfer	2.47	445.1→410.0	80	26
						445.1→426.8	80	17
	Tetracycline-D_6 (IS)	TC-D_6		TRC	2.46	451.3→416	65	26
						451.3→114	65	16

Class	Compound	Abbr	CAS	Supplier	R.T. (min)	MRM-transitions	Fragmentor (V)	CE[d] (eV)
**Fluoroquinolones**		FQs				331.9→314	95	26
	Ciprofloxacin	CFX	85721-33-1	Dr.Ehrenstorfer	2.14	331.9→288.1	95	24
						340.1→322	95	30
	Ciprofloxacin-D_8 (IS)	CFX-D_8		Dr.Ehrenstorfer	2.12	340.1→296.1	95	26
						358→340.1	90	31
	Danofloxacin	DAN	112398-08-0	Dr.Ehrenstorfer	2.64	358→314	90	25
						400.2→356	90	24
	Difloxacin	DIF	98106-17-3	Dr.Ehrenstorfer	3.96	400.2→381.9	90	27
						360→316.2	100	27
	Enrofloxacin	EFX	93106-60-6	Dr.Ehrenstorfer	2.9	360→342	100	28
						365.1→321.1	70	36
	Enrofloxacin-D_5 (IS)	EFX- D_5		Dr.Ehrenstorfer	2.87	365.1→347.1	70	30
						370.1→326	115	25
	Fleroxacin	FL	79660-72-3	Dr.Ehrenstorfer	1.91	370.1→268.9	115	36
						320→301.8	120	26
	Norfloxacin	NFX	70458-96-7	Dr.Ehrenstorfer	1.93	320→276	120	23
						351.8→265	90	30
	Lomefloxacin	LFX	98079-51-7	Dr.Ehrenstorfer	2.52	351.8→308	90	22
						363→72	95	26
	Marbofloxacin	MAR	115550-35-1	Dr.Ehrenstorfer	1.64	363→319.9	95	21
						362→261	124	35
	Ofloxacin	OFX	82419-36-1	Dr.Ehrenstorfer	1.97	362→318.1	124	26
						334→290	120	26
	Pefloxacin	PEF	70458-92-3	Dr.Ehrenstorfer	2.59	334→316	120	26
						386.1→368	140	27
	Sarafloxacin	SAR	98105-99-8	Dr.Ehrenstorfer	3.79	386.1→342.1	140	26
						263.3→129.0	68	27
	Carbadox	CAR	1791337	Dr.Ehrenstorfer	1.51	263.3→231.1	68	17
**Macrolides**		MLs						
	Azithromycin	ATM	830905-01-5	Dr.Ehrenstorfer	7.59	749.5→158.2	64	40
						749.5→591.5	64	30
	Clarithromycin	CTM	81103-11-9	Dr.Ehrenstorfer	7.59	748.9→158.2	95	37
						748.9→590.6	95	29
	Erythromycin-H_2O	ETM-H_2O	23893-13-2	TRC	7.44	716.6→158.3	85	42
						716.6→558.5	85	21
	Erythromycin-^{13}C–D_3 (IS)	ETM-^{13}C-D_3		TRC	7.44	720.7→162.4	97	42
						720.7→562.5	97	22
	Leucomycin	LCM	1392-21-8	Dr.Ehrenstorfer	7.46	772.7→109.1	132	63
						772.7→174.2	132	44
	Oleandomycin	ODM	3922-90-5	Dr.Ehrenstorfer	7.67	689→158.1	100	42
						689→544	100	28
	Roxithromycin	RTM	80214-83-1	Dr.Ehrenstorfer	7.63	837.8→158.1	102	47
						837.8→679.8	102	29
	Tylosin	TYL	1401-69-0	Dr.Ehrenstorfer	7.26	916.7→174.3	141	55
						916.7→772.4	141	41

Class	Compound	Abbr	CAS	Supplier	R.T. (min)	MRM-transitions	Fragmentor (V)	CE[d] (eV)
**Ionophores**		IPs						
	Salinomycin	SAL	53003-10-4	Dr.Ehrenstorfer	9.99	773.6→431.4	160	68
						773.6→265.2	160	70
	Narasin	NAR	55134-13-9	TRC	10.2	787.7→431.6	175	69
						787.7→531.5	175	62
	Monensin	MON	17090-79-8	Dr.Ehrenstorfer	10	693.5→675.7	170	52
						693.5→479.4	170	70
**aminocoumarins**	Novobiocin	NOV	303-81-1	Dr.Ehrenstorfer	17.3	613.6→189.1	145	24
						613.6→133		72
**lincosamides**	Lincomycin	LIN	154-21-2	Dr.Ehrenstorfer	1.23	407.5→126.1	80	32
						407.5→359.1	80	26
	Lincomycin-D_3 (IS)	LIN-D_3		TRC	1.23	410.6→129.1	80	30
						410.6→362.2	90	25
**ESI-**								
**chloramphenicol derivatives**								
	Florfenicol	FF	73231-34-2	Dr.Ehrenstorfer	2.92	355.7→335.8	60	12
						355.7→184.5	60	25
	Chloramphenicol	CAP	154-75-2	Dr.Ehrenstorfer	3.63	320.8→152	40	21
						320.8→256.8	40	15
	Chloramphenicol-D_5 (IS)	CAP-D_5		Dr.Ehrenstorfer	3.61	325.7→156.8	55	23
						325.7→261.8	55	16

[a] Abbreviation. [b] Retention time. [c] The selected ions for the antibiotic compounds were $[M+H]^+$, except ionophore compounds ($[M+Na]^+$), florfenicol and chloramphenicol ($[M-H]^-$). The underlined MRM transitions were used for quantification. [d] Collision energy. [e] Internal standard.

International Journal of
Environmental Research and Public Health

MDPI

Table S2. Concentrations of antibiotics in ponds around Lake Guchenghu (ng/L).

Analytes	Fre.[a](%)			Max [b]			Min [c]			Med [d]			Mean		
	spring	summer	autumn	spring	summer	autumn	spring	summer	autumn	spring	summer	autumn	spring	summer	autumn
SAs	80	100	100	79.9	672	431	ND [e]	21.7	11.5	42.0	117	169	39.6	198	175
SMX	40	20	20	17.5	21.7	6.40	ND	ND	ND	ND	ND	ND	6.00	4.34	1.28
SDZ	40	80	80	63.2	654	430	ND	ND	ND	ND	115.2	169	24.5	187	169
TMP	60	60	40	24.5	17.1	15.8	ND	ND	ND	4.40	3.20	ND	9.12	6.82	4.18
MLs	100	100	100	405	2530	313	2.10	1.30	1.90	9.70	417	201	89.2	691	166
RTM	0	0	40	ND	ND	0.20	ND	ND	ND	ND	ND	ND	ND	ND	0.05
LCM	20	0	0	8.00	ND	ND	ND	ND	ND	ND	ND	ND	1.60	ND	ND
CTM	0	60	20	ND	76.2	0.05	ND	ND	ND	ND	3.20	ND	ND	17.1	0.01
ETM-H_2O	100	100	100	405	2450	312	1.10	1.30	1.90	2.10	411	201	82.8	674	165
ATM	20	0	20	24.2	ND	5.60	ND	ND	ND	ND	ND	ND	4.84	ND	1.12
CFX	40	0	0	46.5	ND	ND	ND	ND	ND	ND	ND	ND	16.8	ND	ND
MON	80	80	60	326	328	2.45	ND	ND	ND	3.60	6.6	0.4	102	82.5	0.72
FF	60	100	60	161	248	261	ND	21.0	ND	84.0	141	56.0	78.0	138	81.2
LIN	80	80	80	4.40	2.80	2.30	ND	ND	ND	1.40	1.90	0.80	2.02	1.58	0.94
ALL	100	100	100	558	3520	1000	148	190	14.5	365	686	429	328	1110	423

a Frequency of detection of each antibiotic in all surface water samples

b Maximum concentration

c Minimum concentration

d Median concentration

e Not detected

International Journal of
Environmental Research and Public Health

Table S3. Canonical correspondence analysis of the antibiotic concentrations and environmental parameters in crab ponds

Axes		1	2	3	4	Total variance
Eigenvalues	:	0.195	0.104	0.072	0.013	1.000
Species-environment correlations	:	0.666	0.674	0.765	0.443	
Cumulative percentage variance						
of species data	:	19.5	29.9	37.2	38.5	
of species-environment relation	:	50.8	77.8	96.5	100.0	
Sum of all eigenvalues						1.000
Sum of all canonical eigenvalues						0.385

© 2018 by the authors. Submitted for possible open access publication under the terms and conditions of the Creative Commons Attribution (CC BY) license (http://creativecommons.org/licenses/by/4.0/).
